# Current Issues and Perspectives in Patients with Possible Sepsis at Emergency Departments

**DOI:** 10.3390/antibiotics8020056

**Published:** 2019-05-07

**Authors:** Ioannis Alexandros Charitos, Skender Topi, Francesca Castellaneta, Donato D’Agostino

**Affiliations:** 1 Department of Emergency/Urgency, National Poisoning Center, Riuniti University Hospital (OO.RR.) of Foggia, 71122 Foggia, Italy; icharitos@ospedaliriunitifoggia.it; 2 School of Technical Medical Sciences, University A. Xhuvani, Elbasan 3001, Albania; skender.topi@uniel.edu.al; 3 School of Medicine, University of Foggia, 71122 Foggia, Italy; fra_c@live.com; 4 Department of Emergency and Organ Transplantation (DETO), School of Medicine, University of Bari A. Moro, 70124 Bari, Italy

**Keywords:** sepsis, sepsis management, SOFA score, laboratory diagnosis, antibiotics, probiotics, plant extract

## Abstract

In the area of Emergency Room (ER), many patients present criteria compatible with a SIRS, but only some of them have an associated infection. The new definition of sepsis by the European Society of Intensive Care Medicine and the Society of Critical Care Medicine (2016), revolutionizes precedent criteria, overcoming the concept of SIRS and clearly distinguishing the infection with the patient’s physiological response from the symptoms of sepsis. Another fundamental change concerns the recognition method: The use of SOFA (Sequential-Sepsis Related-Organ Failure Assessment Score) as reference score for organ damage assessment. Also, the use of the qSOFA is based on the use of three objective parameters: Altered level of consciousness (GCS <15 or AVPU), systolic blood pressure ≤ 100 mmHg, and respiratory rate ≥ 22/min. If patients have at least two of these altered parameters in association with an infection, then there is the suspicion of sepsis. In these patients the risk of death is higher, and it is necessary to implement the appropriate management protocols, indeed the hospital mortality rate of these patients exceeds 40%. Patients with septic shock can be identified by the association of the clinical symptoms of sepsis with persistent hypotension, which requires vasopressors to maintain a MAP of 65 mmHg, and serum lactate levels >18 mg/dL in despite of an adequate volume resuscitation. Then, patient first management is mainly based on: (1) Recognition of the potentially septic patient (sepsis protocol-qSOFA); (2) Laboratory investigations; (3) Empirical antibiotic therapy in patients with sepsis and septic shock. With this in mind, the authors discuss the most important aspects of the sepsis in both adults and infants, and also consider the possible treatment according current guidelines. In addition, the possible role of some nutraceuticals as supportive therapy in septic patient is also discussed.

## 1. Introduction

Sepsis is one of the emerging diseases worldwide, and it is bound to have an increasingly important impact on health systems because the mortality of these patients during the hospital stay exceeds 40%. Thus, the incidence and mortality of sepsis are constantly increasing, and are also a result of several factors, such as the aging of the population, the increased survival for chronic and neoplastic diseases, the extension of immunosuppressive, and antibiotic therapies [1,2,3,4,5,6].

Severe sepsis can lead to an organ dysfunction or MODS (Multiple Organ Dysfunction Syndrome), caused by the development of the systemic inflammatory response (SIRS) to infection, putting the patient’s survival at risk. This clinical condition, in which the circulatory and metabolic alterations are sufficiently important, lead to the septic shock which must be recognized early and treated promptly [7,8,9]. Fundamental for clinical and therapeutic approach is the use of the SOFA, or Sequential-Sepsis-Related-Organ Failure Assessment Score, for the evaluation of organ damage. For example, hospitalized patients with a SOFA greater than two have a mortality rate of 10% and therefore must be managed with an adequate level of clinical attention and resources [7,8,9,10].

In the area of the ER (emergency department), many patients present criteria compatible with a SIRS, but only some of them have an associated infection. On the other hand, an infection does not always lead to an inflammatory response measurable with the SIRS criteria. In 2016, the new definition of sepsis was published, while at the beginning of 2017, the new *Surviving Sepsis Campaign Guidelines* were published. The new definition is described as follows: “an acute response of the body’s immune system to a severe infection, often resulting in damage to the patient’s own tissues and organs” [2,8,10]. Therefore, attempting to make the recognition of the septic patient more immediate and effective in the early phases of admission at the ER, the concept of qSOFA (quick Sepsis Related Organ Failure Assessment) was introduced. It is based on the use of three objective parameters: 1) Altered level of consciousness or the change of mental status (GSC <15 or AVPU), 2) respiratory rate, and 3) systolic blood pressure. (Table 1). The presence of at least two of these altered parameters, in association with an infection, should prompt the suspicion of sepsis. In these patients, the risk of the mortality is very high, and it is necessary to implement the appropriate clinical management already since the patient is being observed in the ER [8,10,11].

## 2. Recognition and Management of the Septic Patient at the Emergency Room

The management of sepsis in the ER is aimed at optimizing early recognition and promptly initial approach of the septic patient, eliminating or reducing avoidable delays. For these patients, the best practice is a correct clinical approach and early goal directed therapy of sepsis at the emergency room. For this, it is appropriate to have a multidisciplinary expert team that includes at least the following specialists: Emergency room physician, infectious disease specialist, internist, anesthesiologist, an intensive care specialist, and, moreover, the nursing support [8,9]. In addition to age and/or to pre-existing clinical conditions (diabetes, heart failure, COPD, immune suppression etc.), which can worse the clinical course, sepsis is due to various causes and may start from the skin and mucosal tissues, cardiopulmonary tract (heart and vascular diseases, healthcare associated pneumonia, hospital acquired pneumonia, etc.), abdominal district, urinary tract, or, finally, starting causes that remain unknown [8,12,13,14].

**Figure 1:**
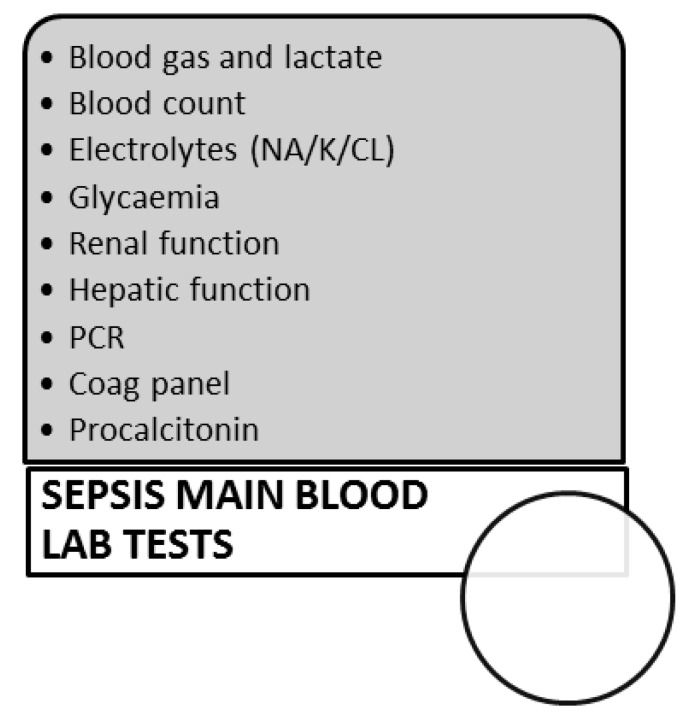
Panel of the blood investigations.

The first approach must start from the use of qSOFA (quick SOFA) at the Emergency Department Triage room for the identification of septic patients with greater risk. The qSOFA is “positive” if at least two of the following criteria are met in the presence of signs of infection: Tachycardia (> 120), SatO2 (<92%), urinary restriction in the last 20 h, state of agitation, and change in mental status (identified as GCS (<15) with cutaneous signs of hypoperfusion. We also emphasize that the patients with modest signs of dysfunction may deteriorate later, highlighting the severity of their condition and the need for an appropriate therapeutic approach [8,10,11]. After vital signs monitoring can be performed an electrocardiogram (EKG), the main blood and urine tests, blood gas measurement (to investigate the severe acidosis with high lactates), procalcitonin (data available in the literature suggest that procalcitonin is a valid aid in the early diagnostic phase, at least within the 12 h following admission), and if available in selected cases, b-D-glucan and galactomannan for the diagnosis of fungal infections [8,10,15,16,17]. Furthermore ultrasound, X-ray of the chest or/and abdomen, or TC scan can be performed according to the symptoms or clinical suspicion. Cultures of blood, urine, respiratory secretions, and if necessary, of the cerebrospinal fluid, must be performed based on the clinical symptoms for the research of the infective focus [10,18,19]. The correct measurement of lactate levels within the first hour after admission of the patient to the ER is specifically associated with better results in the treatment of the disease. In fact, elevated lactic acid in the blood (which indicates an anoxic tissue damage) and a missed renal clearance at two hours identify patients with a severe prognosis *quoad vitam*. (Figure 1). These previously reported procedures can be repeated after three hours if the patient remains in the emergency room observation [9,10,18,20,21].

However, it is not appropriate for the patient with sepsis or septic shock to stay in the ER more than three hours, even if specialized or intensive care occurs. This means that after the first treatment approach through ER management, some patients must be sent to the most suitable department for subsequent care based on the clinical and diagnostic elements. Throughout the patients stay in ER, it is necessary to ensure an adequate level of monitoring and assistance [1,8,19,20,22].

The patients with septic shock can be identified by the association of the clinical symptoms of sepsis with persistent hypotension that requires vasopressor agents to maintain an average blood pressure of 65 mmHg, and that have serum lactate levels >18 mg/dl despite adequate volume resuscitation. If the patient shows conditions of severe hemodynamic instability, then the diuresis must be monitored (also by urinary catheterization), as well as liquids infusions and the management with amino vasopressor agents should be considered [2,10,23].

For the pediatric patient, keeping in mind that the child has an extracellular/intracellular fluid ratio greater than the adult, he/she may be more vulnerable to hypovolemia [24,25,26]. Moreover, the younger is a child, and the greater is the heart rate because it must sustain an appropriate cardiac output. Therefore, cardiac output depends on heart rate, and low cardiac output is related to mortality risk [27,28]. This concept is fundamental to better understand the evaluation of vital parameters in the septic child. Tachycardia is the fundamental mechanism that the septic patient uses to maintain the cardiac output, but for a very young child patient, it is more difficult to maintain a sufficient cardiac output by the increase of the heart rate [29,30]. As a consequence, in the septic young child a heart rate less than 70 or more than 150 beats/min is associated with a greater mortality. So, a hypotensive septic child is already in a phase of decompensated shock. When the heart rate is no longer sufficient, the maintenance of an adequate systemic arterial pressure is guaranteed by peripheral vasoconstriction [26,29]. This means that the pediatric patients septic shock arises later as opposed to adult patients. In clinical practice these concepts explain why the so called “cold shock,” characterized by an increase of peripheral vascular resistances, is more frequent in children, while in adult’s patients the “warm shock” (with decrease of peripheral vascular resistance) prevails [10,29,30].

When the patients condition begins to deteriorate because of respiratory fatigue, we can start noninvasive mechanical ventilation (NIMV) or proceed with intubation and mechanical ventilation, also in accordance to blood gas analysis (arterial hypoxemia ratio: pO_2_/FO_2_ <300) [2,7,9]. It must be mentioned that the pediatric patient has a greater risk of respiratory failure due to a relatively small alveolar surface, combined with a lower pulmonary residual functional capacity [10,30,31,32,33].

Early administration of appropriate antibiotic therapy within 60 min from the identification of the septic condition with appropriate blood cultures is mandatory for effective treatment. Every hour of delay is associated with a significant increase in mortality [10,34,35,36]. However, before starting antibiotic therapies, sample cultures of body fluids other than blood (urine, sputum) should be collected. Therefore, the choice of the initial empirical antibiotic therapy will must be based not only on the anatomical spreads of infection, but also on adequate clinical and epidemiological, with special regard to pharmacodynamic aspects and dosage criteria [34,37,38]. Therefore, in most septic patients, a large number of antibiotics, alone or in combination, can be indicated. Usually, the most used are Amikacin, Amoxicillin plus Clavulanate, Cephazolin, Ceftazidime, Cephtriaxon, Cephtobiprole, Ceftolozam plus Tazobactam, Clarithromycin, Ciprofloxacin, Levofloxacin, Clyndamicin, Daptomycin, Phosphomycin, Gentamycin, Imipenem, Linezolid, Meropenem, Oxacillin, Piperacillin plus Tazobactam, Telavancin, Tigecyclin, Trimethoprim plus Sulfamethoxazole, and Vancomycin. In addition, fungicides such as Fluconazol, Anidulafungin, Capsofungin, and Micafungin must also be considered after positive blood culture for yeast [35,39,40,41,42].

An initial empirical therapy approach must include the use of broad-spectrum antibiotics. It is advisable to use two different classes of antibiotics, for example, the association of penicillin or a third or fourth generation cephalosporin associated to an aminoglycoside [42,43,44]. For example, if the infectious focus is not detected, or a urinary tract or respiratory infection is suspected, antibiotic therapy can be started with cephtriaxon or cephotaxim. If an infection of the central nervous system is suspected, riphampicin or vancomycin can also be added [10,45,46]. In cases of cellulitis or fasciitis, a third-generation cephalosporin with possible association of vancomycin is the best option, while in neutropenic patients, it is reasonable to start with cephtazidim or piperacillin plus tazobactam [47,48,49]. The antifungal therapy should be considered in the immunocompromised patients, diabetics, and for patients with long-lasting fever unresponsive to antibiotic therapy [2,34,35]. In the subsequent phases of patient management, antibiotic therapy must be re-evaluated to proceed with correct pharmacological management based on the results of diagnostic procedures [2,10].

## 3. Conclusions

Sepsis is a complex pathology requiring the rapid and coordinated involvement of different professionals in a multidisciplinary team at the ER department through the perspective of best practice management in the medical emergency room. The clinical management of the patient with sepsis or septic shock must be rapid and effective for an outcome without fatal complications. Empirical antibiotic therapy must be based on the etiology and clinical conditions, as determined by clinical and technical tools [10,34,35]. To date, although many uncertainties and reasons for extensive discussion of definition remain in pathophysiology and clinical management, we can state that scientific evidence and international consensus documents clearly identify some key points for the proper management of sepsis through an early identification, blood investigations, lactate measurement, culture tests before antibiotic therapy, antibiotic therapy as early as possible (at best within 60 min), and early management of hemodynamic by fluid infusion and use of vasopressor agents.

Prospectively, new medical instruments and further investigations about the role of the human microbiome in regulating healthy and pathologic conditions could give us a number of fundamental information about the pathophysiologic mechanisms of the sepsis [50,51,52,53,54]. Moreover, both the arising knowledge on antibiotic activity of plant extracts [55,56,57,58,59,60,61,62] and on the immune modulating effect of probiotics will be helpful to develop most rapid and economic protocols for septic patients [63,64,65,66,67,68,69,70,71,72,73].

## Figures and Tables

**Table 1:** qSOFA (quick Sepsis Related Organ Failure Assessment)

Criteria	Points
Respiratory rate ≥22	1
Change in mental status	1
Systolic blood pressure ≤100	1
